# Interview based malnutrition assessment can predict adverse events within 6 months after primary and revision arthroplasty – a prospective observational study of 351 patients

**DOI:** 10.1186/s12891-018-2004-z

**Published:** 2018-03-15

**Authors:** Christoph Ihle, Christoph Weiß, Gunnar Blumenstock, Ulrich Stöckle, Björn Gunnar Ochs, Christian Bahrs, Andreas Nüssler, Anna Janine Schreiner

**Affiliations:** 1 Siegfried Weller Institute for Trauma Research, BG Trauma Center Tübingen, Eberhard Karls University Tübingen, Schnarrenbergstrasse 95, 72076 Tübingen, Germany; 2 Department of Clinical Epidemiology and Applied Biometry, Eberhard Karls University Tübingen, Silcherstrasse 5, 72076 Tubingen, Germany; 3 Department of Orthopedics and Trauma Surgery, Medical Center, Albert-Ludwigs-University of Freiburg, Faculty of Medicine, Freiburg, Germany

**Keywords:** Malnutrition, Arthroplasty, Interview based assessment, NRS 2002, MNA, SF-MNA

## Abstract

**Background:**

Being at risk for malnutrition can be observed among hospitalized patients of all medical specialties. There are only few studies in arthroplasty dealing with defining and assessing malnutrition as such a potentially risk. This study aims to identify the risk for malnutrition following primary (pAP) and revision arthroplasty (rAP) (1) using non-invasive interview based assessment tools and to analyze effects on clinical outcome (2) and quality of life (3).

**Methods:**

A consecutive series of hospitalized patients of a Department of Arthroplasty at a Level 1 Trauma Center in Western Europe was observed between June 2014 and June 2016. Patients were monitored for being at risk for malnutrition at hospital admission (T1) and 6 months post surgery (T2) by non-invasive interview based assessment tools (NRS 2002, SF-MNA, MNA). Adverse events, length of hospital stay and quality of life (HRQL, SF-36) were monitored.

**Results:**

351 (283 pAP/ 68 rAP) patients were included. At T1, 13.4% (47) / 23.9% (84) / 27.4% (96) and at T2 7.3% (18) / 17.1% (42) / 16.0% (39) of all patients were at risk for malnutrition regarding NRS/SF-MNA/MNA. Prevalence of malnutrition risk was higher in rAP (22.1–29.4%) compared to pAP (11.3–26.9%). Patients being at risk for malnutrition showed prolonged hospitalization (NRS 14.5 to 12.5, SF-MNA 13.7 to 12.4, MNA 13.9 to 12.3 days, *p* < 0.05), delayed mobilization (NRS 2.1 to 1.7, SF-MNA 1.8 to 1.7, MNA 1.9 to 1.7 days), lower values in HRQL and more adverse events.

**Conclusions:**

There is a moderate to high prevalence of risk for malnutrition in arthroplasty that can easily be assessed through interview based screening tools. Being at risk for malnutrition can reduce the clinical outcome following pAP and rAP. Patients with an impaired nutritional status show reduced values in physical and mental aspects of HRQL. Non-invasive interview-based nutritional assessment can predict adverse events in primary and revision total arthroplasty and can therefore help identifying patients at risk before surgery.

**Trial registration:**

The study protocol was approved by the local ethics committee (193/2014BO2) and registered at the German Clinical Trials Register according to WHO standard (DKRS00006192).

## Background

Impaired nutritional status can be observed among hospitalized patients across all medical specialties with prevalence rates between 20 and 61% [1, 2]. Several studies have shown the substantial importance of malnutrition and its impact on clinical results [3, 4]. Higher age as well as disease- and lifestyle associated circumstances are risk factors for developing nutritional deficiencies [5]. It is an increasing problem with relevant consequences regarding medical as well as socio-economic aspects due to higher complication rates and prolonged hospitalization accompanied by demographic changes resulting in higher costs for hospitals and health insurances [3, 6]. The relevance is underlined by an increasing number of publications in the past years, especially the latest ESPEN (European Society for Clinical Nutrition and Metabolism) publications and definitions [7, 8]. Nevertheless, there is still little information concerning the nutritional status of hospitalized orthopedic and trauma patients [9, 10]. The prevalence of malnutrition is estimated between 9 and 39% in orthopedic patients undergoing arthroplasty [9]. Studies so far used controversially discussed invasive biomarkers, e.g. albumin, to show that malnutrition is associated with delayed wound healing, periprosthetic joint infections, prolonged length of hospital stay and re-mobilization as well as increased mortality in patients undergoing total hip and knee replacement [9, 11–14].

There are different screening tools to evaluate patients’ probability of being nutritionally at risk. Depending on the used screening method, the prevalence of malnutrition in geriatric patients suffering hip related fractures ranges between 32 and 60% [15–17] and was analyzed as an independent factor of 12-month mortality [18]. ESPEN recognizes the NRS 2002 as well as the SF-MNA as risk screening tools to be used in hospital, elderly care and community settings [8]. Besides the risk screening tools named above, blood markers for malnutrition like albumin, total leucocyte count and transferrin are in controversial discussion [14, 19–21]. However, the relevance of patient assessment at risk for malnutrition in a large and prospectively evaluated case series comparing NRS 2002, SF-MNA and MNA side-by-side as established non-invasive interview based screening tools in patients undergoing primary and revision arthroplasty documented from the time point of hospital admission following six months after hospital dismissal does not exist so far. In contrast to other medical specialties, malnutrition in arthroplasty is partially underreported and needs further evidence, especially regarding evaluation, clinical application of scoring systems and health related quality of life.

## Methods

### Study population

The study protocol was approved by the local ethics committee (193/2014BO2) and registered at the German Clinical Trials Register according to WHO standard (DKRS00006192). Written consent of the ethics committee of the medical university of Tübingen is available. All included subjects gave their informed written consent. The study was conducted according to the guidelines for reporting of observational studies in Epidemiology (STROBE) [22].

A consecutive series of hospitalized patients of a supra-regional Department of Arthroplasty at a Level 1 Trauma Center in Western Europe was observed between June 2014 and June 2015. All patients who agreed to participate in the study and were hospitalized for more than two nights were included. Minors, patients suffering from dementia, with insufficient knowledge of the study language and incapability to answer the questions due to severe health conditions were excluded. Patients at the Intensive Care Unit were generally excluded as being defined as patients with severe health conditions.

The patients were checked for their nutritional status respectively for being at risk for malnutrition at the time of their hospital admission one day prior to operation (T1). Three interview-based, internationally approved and well-established assessment tools were used for the assessment: The Nutritional Risk Screening (NRS 2002), the Mini Nutritional Assessment (MNA) as well as the Short Form MNA (SF-MNA) were administered to all patients. To evaluate the generic quality of life, SF-36 questionnaire was used.

All comorbidities and further information concerning patients’ health and general conditions were collected using a standardized protocol. The diagnoses were self-reported and depended on previously diagnoses and/or taken medication. Adverse events and further general patient data were captured out of our hospital information system after discharge. These data were used for further statistical evaluation as well. All interview based assessments were repeated 6 months after surgery (T2). The assessment was carried out by eight independent observers. Observer bias was avoided by preceding standardized observer training for two weeks in groups of two with frequently changing partners.

### Nutritional risk screening (NRS 2002)

Nutritional Risk Screening (NRS 2002) by Kondrup et al. is an international well-established interview-based assessment tool describing the risk for malnutrition in hospitalized patients [23]. It has been used in several clinical trials of different medical fields so far and is recommended by the European Society for Clinical Nutrition and Metabolism (ESPEN) [7]. Being nutritionally at risk is defined as a NRS-Score ≥ 3 whereas a regular nutrition state is implied by a NRS < 3. Besides registering weight loss within the last 3 months, patient age and the severity of illness are included in this score [7].

### Mini nutritional assessment (MNA)

The Mini Nutritional Assessment (MNA) was developed and validated by the Nestlé Nutrition Institute during studies between 1991 and 1993 in the United States of America and France [24]. Briefly, the application of the MNA is recommended for elderly patients over 65 years. We applied it to all patients of our study. It can be downloaded for free on the internet (http://www.mna-elderly.com). It is a well-established screening tool for malnutrition and has been used in several clinical trials so far. Being at risk of malnutrition is implied by MNA ≤ 11 points. The score includes amongst others Body Mass Index (BMI), behavior concerning food and recent weight loss.

### Short form mini nutritional assessment (SF-MNA)

The SF-MNA (Mini Nutritional Assessment Short-Form) was also developed by Nestlé and is available at the internet (http://www.mna-elderly.com) [25, 26]. Briefly, being nutritionally at risk is defined between a range of 0–7 points. The SF-MNA is applied to elderly patients over 65 years. It is also a well-established assessment tool and of easy and fast application with good accuracy for assessing risk for malnutrition. Next to weight loss within the last 3 months, BMI, mobility and severity of illness are included in this score.

### Health related quality of life (SF-36 questionnaire)

To describe Health Related Quality of Life (HRQL) the SF-36 questionnaire was used [27]. We used the interview-based version of this tool consisting of 36 questions. Based on these questions’ values, eight scales describing physical and mental health can be assessed. The physical part is represented by Physical Functioning (PF), Role Physical (RP), Bodily Pain (BP) and General Health (GH). Vitality (VT), Social Functioning (SF), Role Emotional (RE) and Mental Health (MH) describe the mental aspect of HRQL. Usually regular scoring is performed first (RS, 0–100 points). Physical and mental component summary scores (PSC and MCS) can be achieved by transformation of the summary scores and is a widespread method [28–30].

### Clinical outcome and patient data

Clinical outcome was described through adverse events, length of stay (LOS) and mobilization after primary or revision arthroplasty. Postoperative mobilization was defined as safe walking on crutches out of bed. This information was captured out of the rehabilitation protocol of the clinical information system. Patient data (comorbidities, number of daily medication etc.) were captured out of the digital clinical information system after patients’ discharge.

### Adverse events

Adverse events were monitored at two points of time - during hospital stay and at T2 for the period of 6 months following primary or revision arthroplasty excluding adverse events having already occurred during hospitalization. All adverse events weighted equally. Major adverse events were defined as death, infections, wound healing disorders, further operations and thrombosis. Minor adverse events were postoperative anemia and postoperative electrolyte imbalance with therapeutic necessity and delayed removal of drains more than two days following surgery.

### Statistics

Evaluated scales and scores were quantitative or semiquantitative parameters in the present study. They were described as mean and standard deviation, minimum and maximum, as well as quartiles. Normality was tested with the Kolmogorov-Smirnov-Test. Nominally and ordinally scaled values were displayed in counts and percentages. Contingency tables were used to compare two of each of these values and the chi-square-test was applied for testing association. The drop-out analysis was performed with the t-test for independent samples for the variables age and BMI and with cross-tabulations with chi-quadrat-statistics for the variables sex, alcohol consumption and understanding of the study language. The tests were two sided with a significance level of 5%. An alpha adjustment for multiple testing was not applied. The results were interpreted accordingly. Statistical analysis was performed with IBM SPSS Statistics 23 (SPSS Inc., IBM Company Chicago, IL, USA) and with JMP 13 (SAS, SAS Institute, Cary, NC, USA).

## Results

### Patient characteristics and general information

**Fig. 1:**
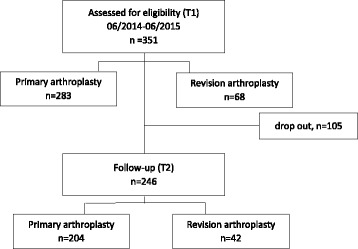
Patient flow chart according to STROBE standards

A patient flow chart is presented in Fig. 1. 351 arthroplasty patients were included in the present study for statistical analysis*.* The whole group can be divided in the subgroup of primary arthroplasty (pAP, *n* = 283) and revision arthroplasty (rAP, *n* = 68). There was no relevant statistical difference in patient age between the subgroups with a mean age of all patients of 67.9 ± (28–91) years (*p* = 0.69). Women outnumbered men in both subgroups. According to the World Health Organization (WHO 2004) our study population can be classified as predominantly pre-obese regarding BMI (25.0–29.9) without noticeable statistical differences regarding the subgroups pAP and rAP (*p* = 0.61).

### Follow-up

Addressing an expected loss to follow-up, a drop-out-analysis was performed. Based on the drop-out-analysis of all eligible and assessed 351 patients (T1), a follow-up of 70,1% (*n* = 246) was achieved without any relevant statistical differences between the group with a total follow-up (T2) and the group lost to follow-up after T1 regarding nutritional status, sex, BMI and age. Nevertheless, there was a statistical difference regarding “language skills of the study language”. Statistically significant more patients who quit the study after T1 showed reduced skills of the study language. In detail, the follow-up was 72,1% (*n* = 204) for the primary arthroplasty group (pAP) respectively 61,8% (*n* = 42) for the revision arthroplasty group (rAP). The question of drop out needn’t to be included in further analysis according to missing statistical differences between the two groups.

### Prevalence of malnutrition in primary and revision arthroplasty

At the time of hospital admission (T1), 13.4% (*n* = 47) / 23.9% (*n* = 84) / 27.4% (*n* = 96) of all patients were at risk for malnutrition according NRS, SF-MNA, and MNA, respectively.

Among pAP patients 11.3% (*n* = 32) / 23.3% (*n* = 66), and 26.9% (*n* = 76) were at risk for malnutrition according to NRS, SF-MNA, and MNA respectively. In the group of rAP patients 22.1% (*n* = 15), 26.5% (*n* = 18), and 29.4% (*n* = 20) were at risk for malnutrition according to NRS, SF-MNA, and MNA respectively. Based on the three different tools we found the highest prevalence of malnutrition when applying the MNA assessment tool in patients undergoing revision arthroplasty.

At the time of follow-up (T2), we found that among all study subjects 7.3% (n = 18), 17.1% (*n* = 42), and 16.0% (*n* = 39) were still at risk for malnutrition regarding NRS, SF-MNA, and MNA respectively.

Among pAP patients, we found at T2 5.9% (*n* = 12), 16.2% (*n* = 33), and 15.4% (*n* = 31) were at risk for malnutrition according to NRS, SF-MNA, and MNA respectively. In the group of rAP patients 14.3% (*n* = 6), 21.4% (*n* = 9), and 19.1% (*n* = 8) according to the three assessment tools respectively. At T2 the highest prevalence of malnutrition risk was assessed by SF-MNA tool. Nevertheless, there was a clear reduction of the risk of malnutrition within six months (from T1 to T2) in all groups. This was not statistically significant, most likely due to small sample sizes.

### Malnutrition and quality of life

**Fig. 2:**
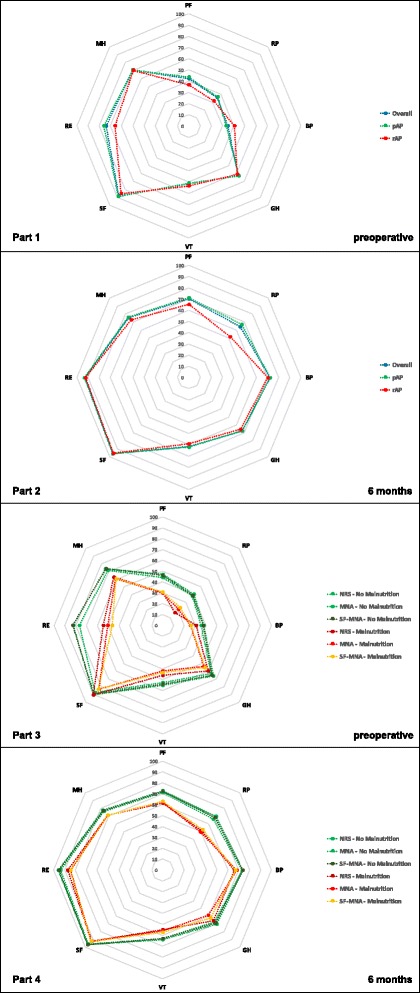
Health related quality of life in primary and revision arthroplasty comparing different nutritional status. Part 1 and 2: Figure two part one and two describe the values of health related quality of life at hospital admission and six months following surgery in all eight SF-36 dimensions comparing primary arthroplasty (pAP), revision arthroplasty (rAP) and the whole study population (overall). A pre- to postoperative improvement of HRQL can be found according to all eight dimensions. Part 3 and 4: Part three and four show the eight SF-36 dimensions of the whole study population according to all three nutritional scores comparing the nutritional status (Malnutrition vs. No Malnutrition) at hospital admission and six months following surgery. HRQL is preoperatively decreased. Patients being at risk for malnutrition before surgery show statistically significant lower values in physical (PF, RP, BP, GH) as well as mental dimensions (SF, RE, MH). In comparison to the preoperative SF-36 results, the values of the patients with risk for malnutrition are adjusting to the values of the patients with a regular nutritional status. PF: Physical Functioning; RP: Role Physical; BP: Bodily Pain; GH: General Health; VT: Vitality; SF: Social Functioning; RE: Role Emotional; MH: Mental Health; pAP: Primary Arthroplasty; rAP: Revision Arthroplasty; HRQL: Health related quality of life

HRQL for the whole study population is presented in Fig. 2 at both time points of evaluation. Part one shows the values of HRQL in all eight dimensions for the whole study population as well as the two subgroups pAP and rAP at T1. Part two describes the development of HRQL six months after surgery for the above mentioned three groups. A significant pre- to postoperative (T1 to T2) improvement of HRQL can be found in all eight dimensions for the overall collective (*p* < 0.001). Part three shows the eight SF-36 dimensions according to the nutritional scores respectively the nutritional status (with or without risk for malnutrition) at T1. HRQL is preoperatively decreased for both nutritional groups (with or without risk for malnutrition), especially for PF, RP, BP, VT and RE. Moreover, patients being at risk for malnutrition before surgery show statistically significant lower values - according mainly to SF-MNA and MNA - in all physical (PF, RP, BP, GH) as well as mental dimensions (SF, RE, MH) in comparison to those with a regular nutritional status. Part 4 describes HRQL according to patients’ nutritional status postoperatively. All 8 dimensions show an increase for both nutritional groups (with or without risk for malnutrition) after surgery. In comparison to the preoperative SF-36 results, the values of the patients with risk for malnutrition are adjusting to the values of the patients with a regular nutritional status, but BP and GH are still statistically significant lower for patients being at risk for malnutrition (*p* = 0.02 respectively *p* = 0.001 according to SF-MNA) than for patients without being at risk.

In summary, patients being at risk for malnutrition show lower values in each dimension of HRQL before and after surgery in comparison to patients with a regular nutritional status regarding the physical as well as mental aspects of SF-36. Improvements after arthroplasty can be found in both nutritional groups in all dimensions with a persisting but closer gap between the values of patients at risk for malnutrition and those with a regular nutritional status.

#### HRQL in relation to nutritional scores

Regarding the relation of nutritional scores at T1 und SF-36 at T2, the MNA shows a statistically significant effect in almost all dimensions (p: PF 0.02 / RP 0.016 / GH 0.004 / VT 0.022 / RE 0.006 / MH 0.044). The SF-MNA unfolded a statistical significance for RE (*p* = 0.018) and MH (*p* = 0.04). The NRS showed no statistically significant difference in any dimension.

### Clinical outcome

#### LOS and mobilization

The average LOS was 12.7 ± 3.65 (6–33) days. Mean LOS was 12.1 ± 2.7 (6–31) days for the pAP group respectively 15.31 ± 5.52 (8–33) days for the rAP group. Mobility in all patients was achieved 1.74 ± 0.79 (1–7) days after arthroplasty was performed. It took 1.7 ± 0.6 (1–5) days in the pPA group respectively 2.12 ± 1.11 (1–7) days in the rAP group until postoperative mobilization could be obtained out of bed.

All Patients assessed at T1 at risk for malnutrition showed a highly statistically significant prolonged length of hospital stay for all assessment tools (*p*-value NRS, SF-MNA, and MNA: 0.006, 0.001, and 0.0001, respectively). In parallel, we found that mobilization after surgery was equally statistically significant delayed compared to patients with a regular nutritional status according to NRS (*p* = 0.012). LOS and mobilization divided according to the different nutritional scores are shown in Table 1.

**Table 1:** Length of hospital stay (LOS) and Mobilization in relation to NRS, SF-MNA and MNA

NRS				
Risk for malnutrition / no risk		+	–	p
all	Hospitalization (LOS, days)	14.5 ± 5.3	12.5 ± 3.2	0.006
all	Mobilization (days after surgery)	2.1 ± 1.0	1.7 ± 0.7	0.012
SF-MNA		T1		
Risk for malnutrition / no risk		+	–	p
all	Hospitalization (LOS, days)	13.7 ± 3.9	12.4 ± 3.5	0.001
all	Mobilization (days after surgery)	1.8 ± 0.9	1.7 ± 0.7	0.267
MNA		T1		
Risk for malnutrition / no risk		+	–	p
all	Hospitalization (LOS, days)	13.9 ± 4.3	12.3 ± 3.3	0.001
all	Mobilization (days after surgery)	1.9 ± 1.0	1.7 ± 0.6	0.189

#### Incidence of adverse events in primary and revision arthroplasty during hospitalization

The incidence and subdivision of adverse events regarding all patients and regarding pAP and rAP is demonstrated in Table 2. Adverse events divided according to the different nutritional scores and according the above mentioned groups are shown in Table 3. Patients being at risk for malnutrition according to interview based assessment tools showed a higher incidence of all adverse events (minor and major) before and after arthroplasty compared to patients with a regular nutritional status. The highest prevalence of adverse events was found when patients were assessed by the NRS assessment tool.

**Table 2:** Incidence of major (grey) and minor adverse events in primary and revision arthroplasty

	all		pAP		rAP	
No adverse events T1	62.1% (218)		66.4% (188)		44.1% (30)	
No adverse events T2	89.1% (219)		91.2% (186)		78.6% (33)	
Adverse events T1	37.9% (133)		33.6% (95)		55.9% (38)	
Adverse events T2	10.9% (27)		8.8% (18)		21.4% (9)	
Infection T1	3 / 351	0.9%	1 / 283	0.4%	2 / 68	2.9%
Infection T2	8 / 246	3.3%	5 / 204	2.5%	3 / 42	7.1%
Wound healing disorder T1	3 / 351	0.9%	2 / 283	0.7%	1 / 68	1.5%
Wound healing disorder T2	2 / 246	0.8%	2 / 204	1.0%	0 / 42	0.0%
Further operations T1	5 / 351	1.4%	2 / 283	0.7%	3 / 68	4.4%
Further operations T2	1 / 246	0.4%	1 / 204	0.5%	0 / 42	0.0%
Thrombosis T1	1 / 351	0.3%	0 / 283	0.0%	1 / 68	1.5%
Thrombosis T2	2 / 246	0.8%	2 / 204	1.0%	0 / 42	0.0%
Minor adverse events T1	121 / 351	34.5%	90 / 283	31.8%	31 / 68	45.6%
Minor adverse events T2	14 / 246	5.7%	8 / 204	3.9%	6 / 42	14.3%

**Table 3:** All Adverse events in relation to NRS, SF-MNA and MNA

NRS		T1			T2		
Risk for malnutrition / no risk		+	–	p	+	–	p
all	Adverse events	53.2% (25)	35.5% (108)		22.6% (7)	9.3% (20)	
	No adverse events	46.8% (22)	64.5% (196)	0.02	77.4% (24)	90.7% (195)	0.03
	*n total*	*47*	*304*		*31*	*215*	
pAP	Adverse events	40.6% (13)	32.7% (82)		13.6% (3)	8.2% (15)	
	No adverse events	59.4% (19)	67.3% (169)	0.37	86.4% (19)	91.8% (167)	0.40
	*n total*	*32*	*251*		*22*	*182*	
rAP	Adverse events	80.0% (12)	49.1% (26)		44.4% (4)	15.2% (5)	
	No adverse events	20.0% (3)	50.9% (27)	0.03	55.6% (5)	84.8% (28)	0.06
	*n total*	*15*	*53*		9	*33*	
SF-MNA		T1			T2		
Risk for malnutrition / no risk		+	–	p	+	–	p
all	Adverse events	46.4% (39)	35.2% (94)		17.9% (10)	8.9% (17)	
	No adverse events	53.6% (45)	64.8% (173)	0.06	82.1% (46)	91.1% (173)	0.06
	*n total*	*84*	*267*		*56*	*190*	
pAP	Adverse events	39.4% (26)	31.8% (69)		12.8% (6)	7.6% (12)	
	No adverse events	60.6% (40)	68.2% (148)	0.25	87.2% (41)	92.3% (145)	0.28
	*n total*	*66*	*217*		*47*	*157*	
rAP	Adverse events	72.2% (13)	50.0% (25)		44.4% (4)	15.2% (5)	
	No adverse events	27.8% (5)	50.0% (25)	0.10	55.6% (5)	84.8% (28)	0.06
	*n total*	*18*	*50*		*9*	*33*	
MNA		T1			T2		
Risk for malnutrition / no risk		+	–	p	+	–	p
all	Adverse events	49.0% (47)	33.7% (86)		17.5% (11)	8.7% (16)	
	No adverse events	51.0% (49)	66.3% (169)	0.01	82.5% (52)	91.3% (167)	0.06
	*n total*	*96*	*255*		*63*	*183*	
pAP	Adverse events	43.2% (33)	29.9% (62)		15.1% (8)	6.6% (10)	
	No adverse events	56.8% (43)	70.1% (145)	0.03	8.9% (45)	93.4% (141)	0.06
	*n total*	*76*	*207*		*53*	*141*	
rAP	Adverse events	70.0% (14)	50.0% (24)		30.0% (3)	18.8% (6)	
	No adverse events	30.0% (6)	50.0% (24)	0.13	70.0% (7)	81.2% (26)	0.45
	*n total*	*20*	*48*		*10*	*32*	

## Discussion

Our study could show a moderate to high prevalence of 13 to 27% being at risk for malnutrition in arthroplasty patients prior to surgery. The main findings of the present study were that being at risk for malnutrition results in suboptimal postoperative clinical outcome. Based on our data, prolonged hospitalization, delayed mobilization after surgery, more adverse events and reduced health related quality of life have to be expected in these patients. Non-invasive interview-based nutritional assessment can predict adverse events in primary and revision total arthroplasty and can therefore help identifying patients at risk before surgery.

The aim of this study was to determine the prevalence of risk for malnutrition in hospitalized patients undergoing primary and revision arthroplasty and to analyze its clinical effects up to 6 months after surgery. Moreover, we wanted to evaluate the relation between impaired nutritional status and health related quality of life as well as to determine which screening tool is useful in daily clinical practice. Based on our data, being at risk for malnutrition according to NRS-, SF-MNA-, and MNA assessment results clearly in suboptimal clinical outcomes. Independently of the assessment tool used, the risk for malnutrition was associated with prolonged hospitalization, more wound healing disorders, more adverse events and reduced health related quality of life compared to patients with a regular nutritional status. NRS tool identified patients being at risk for malnutrition that were associated with higher rates of adverse events superior compared to the other used tools.

As the risk for malnutrition was significantly reduced in all patients, as well as in pAP and rAP after operative treatment, arthroplasty obviously reduced the pain level, improved the mobility, and ameliorated the initial impaired nutritional status. Nevertheless, our data shows that being at risk for malnutrition is a relevant risk factor for several aspects of a diminished clinical outcome that seems to be undervalued so far in clinical routine. Non-invasive interview-based nutritional assessment can predict adverse events in primary and revision total arthroplasty and can therefore help identifying patients at risk before surgery. To our knowledge, this is the first study analyzing these results for primary as well as revision arthroplasty with the focus on interview based assessment and HRQL in a prospective and huge cohort. The moderate to high prevalence of 13–27% of risk for malnutrition found in our study corresponds with the predominant data presented in a recent review which reports a scope from 9 to 39% with other studies showing incidences as high as 50% [9]. Thomas et al. reported a prevalence of 24.1% of patients at risk for malnutrition according to NRS in elective surgery [2]. This is similar to the finding of Nicholson et al. who defined malnutrition by albumin levels and total lymphocyte count (TLC). The authors concluded from their data a prevalence of 30% in elective THA compared to 86% in trauma THA of malnutrition [20].

Jensen et al. and Golladay et al. reported delayed wound healing, persistent drainage and prosthetic joint infection in orthopedic patients as the most common complications in association with malnutrition [9, 11]. Other studies reviewing and/or defining malnutrition by low transferrin, total lymphocyte count or albumin also describe the following associated complications in arthroplasty: wound complications, extended length of stay and periprosthetic joint infection [20, 31, 32]. Besides, re-admission and further complications such as postoperative hematoma, seroma, renal complications and pneumonia are described [31]. All these findings are in line with our results. As far as we know there has not been an evaluation of HRQL in arthroplasty patients with an impaired nutrition status so far. We could recently show that traumatology and orthopedic patients show lower values in each dimension of SF-36 [16]. Our current results confirm these findings especially for arthroplasty patients. The applied assessment tools in our study have proven to be useful and easy to use according to our data. We and others compared various nutritional assessment tools in elderly trauma patients (NRS, SF-MNA and MUST) with hip fractures [15, 33]. Koren-Hakim et al. reported that only the SF-MNA could predict readmission and mortality besides detecting malnutrition while the other two assessment tools did not [33]. Considering our results, it becomes obvious that the application of different nutritional scores unfolds with similar general and comparable results but with emphasis on different key aspects. In the opinion of the authors, there is an advantage in some scores assessing the following aspects in patients undergoing arthroplasty.

- Risk of malnutrition: MNA, SF-MNA superior to NRS
- Mobilization: NRS
- Adverse events: NRS superior to MNA and SF-MNA
- HRQL: MNA superior to SF-MNA

There are some limitations of the present study. One limitation is the high drop out rate of our cohort. To avoid statistical bias and misinterpretation of the data additional drop out analysis was carried out. More patients, in the group that did not finish the whole assessment, showed reduced skills of the study language. In our opinion, this is the main reason for a drop out rate of nearly 30%. Because of missing statistical differences this aspect was not included in further statistical analysis 6 months after surgery. In contrast to the majority of the few available studies investigating malnutrition in arthroplasty, biomarkers such as Albumin and Pre-Albumin were not quantified in our study. On the one hand, the discussion concerning the use of biomarkers in this context is not yet concluded. Morey et al. recently put into question the values of serum albumin level and TLC as a surrogate marker of malnutrition for predicting wound complications in total knee arthroplasty [21]. In contrast, Nelson et al. described an association of low albumin levels with complications after total knee arthroplasty and Yi et al. an association of nutritional biomarkers with acute postoperative infection [34, 35]. On the other hand, there are no studies so far that concentrate on the evaluation of being at risk for malnutrition by validated and widespread used non-invasive scoring systems in primary as well as in revision arthroplasty. The applied assessment tools in our study however have been approved several times in different medical fields and are globally recommended to identify patients being at risk for malnutrition in a non-invasive way [36]. Interview based assessment is an easy to handle tool and can be well integrated into daily clinical setting [15]. Based on our data and in agreement with studies in trauma and other medical specialties, NRS, SF-MNA and MNA are valuable tools for assessing patients at risk for malnutrition and may be used to predict clinical outcomes and HRQL [36, 37]. Regarding the use of MNA and SF-MNA there is a limitation concerning patients’ age as these scores are recommended for patients older than 65 years [38, 39]. We used these scores nevertheless, as they are well established screening tools. Furthermore, a majority of 65,8% (*n* = 231) of our patients were older than 65 years. Another point of discussion is that the primary and revision AP groups differ in quantity. This fact corresponds with the distribution of arthroplasty patients in general, but it could be interesting to evaluate a larger patient groups undergoing revision arthroplasty as they show a higher prevalence of risk for malnutrition according to our data. Besides serum markers and standardized scoring tools there are also anthropometric measurements as optional surrogate marker for malnutrition [31, 32]. However, these parameters are at least under debate or questioned to identify marginal or acute nutritional deficiency and might be better used as additional indicators for chronic changes in the nutritional status [31, 32]. In our opinion, a screening tool for malnutrition should be fast, non-invasive, economical, applied to all medical fields of a single clinic, and easy to handle in daily clinical routine. After a first screening for malnutrition by interview-based assessment tools, invasive procedures such as biomarkers and anthropometric measurements can be helpful to give additional information and strengthen the diagnosis.

In summary, our study has clearly shown, that there is a moderate to high prevalence of risk for malnutrition in arthroplasty that can easily be assessed through interview based screening tools. This should be integrated as a standard procedure in clinical routine to be able to evaluate patients’ risk for malnutrition as well as the accompanying risks regarding the clinical outcome already before arthroplasty takes place. Several studies have highlighted the necessity to routinely screen patients to detect their risk for malnutrition [9, 40]. Although there is only 7.3% to 29.4% of all cases that have a risk for malnutrition, all tested assessment tools can clearly identify these cases and initiate a possible perioperative intervention such as using oral nutritional supplements. The precise prediction of cases with a risk for malnutrition has most likely also an economical effect, since only the cases of a risk for malnutrition are subject of an interventional therapy and not all patients. In contrast to general or trauma surgery there are so far no studies evaluating this kind of intervention. Compared to trauma patients there is even the chance to substitute elective arthroplasty patients before surgery. Furthermore, the discussion of the use and significance of biomarkers should be continued.

## Conclusions

The prevalence for being at risk for malnutrition for patients undergoing primary or revision arthroplasty is between 7.3% to 29.4% according to our data. Patients with an impaired nutritional status show reduced values in physical and mental aspects of HRQL. The risk for malnutrition results in suboptimal clinical outcomes regarding especially adverse events, length of stay, mobilization and wound healing. Non-invasive interview-based nutritional assessment can predict adverse events in primary and revision total arthroplasty and can therefore help identifying patients at risk before surgery.

## Abbreviations

ESPEN: European Society for Clinical Nutrition and Metabolism
HRQL: Health Related Quality of Life
LOS: Length of Stay
MNA: Mini Nutritional Assessment
NRS: Nutritional Risk Screening
pAP: Primary Arthroplasty
rAP: Revision Arthroplasty
SF-36: Short Form 36
SF-MNA: Short Form Mini Nutritional Assessment
STROBE: Strengthening the reporting of observational studies in epidemiology
THA: Total Hip Arthroplasty
TKA: Total Knee Arthroplasty
